# Supplementary material for: Trends in underlying causes of death in solid organ transplant recipients between 2010 and 2020: Using the CLASS method for determining specific causes of death
Source: PLoS One. 2022 Jul 25;17(7):e0263210. doi: 10.1371/journal.pone.0263210 (PMC9312393; doi:10.1371/journal.pone.0263210)
Supplement: S1 Table — * Doses are adjusted for pediatric patients according to weight and surface area. ** Kidney transplant includes single or multiple kidney transplants (n = 837) and combined pancreas and kidney transplants (n = 29). *** Liver transplant includes single or multiple liver transplants (n = 459) and combined liver and kidney transplants (n = 17). **** Lung transplant includes single or multiple lung transplants (n = 298) and combined lung and kidney transplants (n = 1). ***** Induction therapy for combined pancreas and kidney transplant recipients consists of Anti-thymocyte globulin and Prednisolone. (DOCX) [file pone.0263210.s001.docx]

| Immunosuppression | Heart transplant | Kidney transplant** | Liver transplant*** | Lung transplant**** |
| --- | --- | --- | --- | --- |
| Standard immunosuppression | Cyclosporine/tacrolimus  AND  Mycophenolate mofetil  AND  Prednisolone | Tacrolimus  AND  Mycophenolate mofetil  AND  Prednisolone | Tacrolimus  AND  Mycophenolate mofetil  AND  Prednisolone | Cyclosporine/tacrolimus  AND  Azathioprine or Mycophenolate mofetil  AND  Prednisolone |
| Induction therapy | Anti-thymocyte globulin  AND  Prednisolone | Basiliximab*****  AND  Prednisolone | None for single or multiple liver transplant recipients.  Basiliximab for combined liver-kidney transplant recipients and recipients with impaired kidney function. | Anti-thymocyte globulin  AND  Prednisolone |
|  |  |  |  |  |
| Antimicrobial prophylaxis |  |  |  |  |
| Preoperative | None | Single dose Cefuroxime 1.5g for adults and 40mg/kg for pediatric patients. | None | None |
| Perioperative | None | None | A single dose of 1000 mg Prednisolone. | Meropenem  AND  Ciprofloxacin  AND  Vancomycin |
| Early postoperative period | Cefuroxime (1.5g/3x day) for first 48 hours  OR  Meropenem (2g/3x day) for first 48 hours for patients with left ventricle assist device. |  | Meropenem (15mg/kg/3x day) during the first five days posttransplant. | Meropenem (1g/3x day)  AND  Ciprofloxacin (400mg/2x day) until c-reactive protein was normalized or for two weeks. |
| Pneumocystis jirovecii prophylaxis | Lifelong Trimethoprim-Sulfamethoxazole. | Trimethoprim-Sulfamethoxazole for 6 months posttransplant. | Trimethoprim-Sulfamethoxazole for 6 months posttransplant. | Lifelong Trimethoprim-Sulfamethoxazole. |
| Additional antifungal prophylaxis | Oral Nystatin mixture (5ml/4x day) in the first month posttransplant. | None | Selected recipients with certain surgical complications, CRRT, fulminant hepatitis or CMV infection received either Micafungin or Anidulafungin. | Nystatin (500.000 IE/4x day) was administered in all lung recipients during the period where daily Prednisolone dose was higher than 10mg.  Patients transplanted before July 2016 received Voriconazole (200mg/2x day) for three months.  Only high-risk recipients transplanted after July 2016) received Posaconazole (300mg/1x day)  AND  Inhalation Amphotericin B (25mg/1x day). |
| CMV management | Monitored through the MATCH programme^[21]^ including regular monitoring during the first year posttransplant and valganciclovir prophylaxis for selected patients based on CMV IgG serostatus of donor and recipient.  All heart recipients received valganciclovir 900mg daily (adjusted for kidney function) for the first three months. | Monitored through the MATCH programme^[21]^ including regular monitoring during the first year posttransplant and valganciclovir prophylaxis for selected patients based on CMV IgG serostatus of donor and recipient.  All kidney recipients received valganciclovir 450mg ever other day for the first three months. | Monitored through the MATCH programme^[21]^ including regular monitoring during the first year posttransplant and valganciclovir prophylaxis for selected patients based on CMV IgG serostatus of donor and recipient.  All liver transplant recipients received valganciclovir or valaciclovir 900mg daily (adjusted for kidney function) for the first three months. | Monitored through the MATCH programme^[21]^ including regular monitoring during the first year posttransplant and valganciclovir prophylaxis for selected patients based on CMV IgG serostatus of donor and recipient.  All lung transplant recipients received valganciclovir 900mg daily (adjusted for kidney function) for the first three months posttransplant. |
