# Supplementary material for: Trends in underlying causes of death in solid organ transplant recipients between 2010 and 2020: Using the CLASS method for determining specific causes of death
Source: PLoS One. 2022 Jul 25;17(7):e0263210. doi: 10.1371/journal.pone.0263210 (PMC9312393; doi:10.1371/journal.pone.0263210)
Supplement: S2 Table — (DOCX) [file pone.0263210.s002.docx]

|  | Data source | Data type | Details |
| --- | --- | --- | --- |
| National data |  |  |  |
|  | MedCom | Biochemistry | Data from all labs in Denmark.  From:   - Capital Region of Denmark, complete since September 2014 - Capital Region of Denmark’s Elektive Laboratorium, complete since 2010 - Region Zealand, complete since February 2014 - North Denmark Region, complete since December 2014 - Region of Southern Denmark, complete since 2013 - Central Denmark Region, complete since 2009 |
|  | Danish Microbiology Database (MiBa) (connection through Laboratoriesvarportalen). | Microbiology | Complete for all hospital departments since 2010 and for Statens Serum Institut from 2013 |
|  | Patobank (connection through Laboratoriesvarportalen) | Pathology | Patobank is complete for pathology procedures and diagnoses for all hospital departments since 1997. Laboratoriesvarportalen is complete with data from Patobank since 2005. |
|  | National Patient Registry (LPR) | Admissions, diagnoses and procedures | Collected since 1977 for all patients treated in the Danish healthcare system. From 2017, only regional data was collected. |
|  | Civil registration System (CRS) | Death and emigration | Collected since 1968 for all Danish residents |
| Regional data |  |  |  |
|  | Labka | Biochemistry | Capital Region of Denmark, complete since 2009 |
|  | RIS | Radiology | Data from following hospitals in the capital region of Denmark: Rigshospitalet, Hvidovre Hospital, Bispebjerg Hospital, Frederiksberg Hospital. Collected since 2005. |
|  | Sundhedsdatabanken | Hospital contacts, diagnoses, procedures and demography | Capital region of Denmark, complete since 2007. |
| Local data |  |  |  |
|  | Management of Post-Transplant Infections in Collaborating Hospitals (MATCH) | Clinical characteristics including gender, date of transplantation, transplant type, number of transplantations and date of birth. | MATCH is a clinical application consisting of an individualized surveillance plan which was developed in 2012/13 to earlier detect and manage virus infections among transplant recipients. All transplant recipients transplanted at Rigshospitalet from 2013 and onwards have been enrolled prospectively as part of clinical routine and monitored[1]. Recipients transplanted between 2010 and 2013 were retrospectively included. |

References

1. Ekenberg C, da Cunha-Bang C, Lodding IP, Sørensen SS, Sengeløv H, Perch M, et al. Evaluation of an electronic, patient-focused management system aimed at preventing cytomegalovirus disease following solid organ transplantation. Transpl Infect Dis. 2020;22(2):e13252. Epub 2020/01/31. doi: 10.1111/tid.13252. PubMed PMID: 31997565.
