# Supplementary material for: Trends in underlying causes of death in solid organ transplant recipients between 2010 and 2020: Using the CLASS method for determining specific causes of death
Source: PLoS One. 2022 Jul 25;17(7):e0263210. doi: 10.1371/journal.pone.0263210 (PMC9312393; doi:10.1371/journal.pone.0263210)
Supplement: S3 Table — National pre-transplant chronic disease data were unavailable from registries after 2017. Comorbidities recorded in regional and national registries are based on diagnosis codes registered by attending physicians in various hospitals as part of patient care and data may therefore be incomplete and imprecise. * Kidney transplant includes single or multiple kidney transplants (n = 837) and combined pancreas and kidney transplants (n = 29). ** Liver transplant includes single or multiple liver transplants (n = 459) and combined liver and kidney transplants (n = 17). *** Lung transplant includes single or multiple lung transplants (n = 298) and combined lung and kidney transplants (n = 1). (DOCX) [file pone.0263210.s003.docx]

| Comorbidities | All transplants | Heart transplant | Kidney transplant* | Liver transplant** | Lung transplant*** |
| --- | --- | --- | --- | --- | --- |
| Diabetes mellitus; N (%) | 262 (18) | 15 (14) | 159 (23) | 51 (14) | 37 (15) |
| Cardiovascular disease; N (%) | 159 (11) | 93 (88) | 48 (7) | 7 (2) | 11 (4) |
| Chronic obstructive lung disease; N (%) | 289 (20) | 13 (10) | 37 (4) | 65 (14) | 182 (74) |
| Connective tissue disease; N (%) | 73 (5) | 1 (1) | 43 (6) | 12 (3) | 17 (7) |
| Liver disease; N (%) | 401 (28) | 1 (1) | 25 (4) | 372 (99) | 3 (1) |
| Cerebrovascular disease; N (%) | 100 (7) | 12 (11) | 73 (11) | 12 (3) | 3 (1) |
| Peripheral arterial disease; N (%) | 85 (6) | 41 (39) | 34 (5) | 5 (1) | 5 (2) |
| Kidney disease; N (%) | 762 (54) | 27 (25) | 688 (99) | 18 (5) | 29 (12) |
| Malignant disease; N (%) | 122 (9) | 3 (3) | 35 (5) | 76 (20) | 8 (3) |
| Total; N (%) | 1418 (100) | 106 (100) | 691 (100) | 376 (100) | 245 (100) |
