# Supplementary material for: Trends in underlying causes of death in solid organ transplant recipients between 2010 and 2020: Using the CLASS method for determining specific causes of death
Source: PLoS One. 2022 Jul 25;17(7):e0263210. doi: 10.1371/journal.pone.0263210 (PMC9312393; doi:10.1371/journal.pone.0263210)
Supplement: S4 Table — * Kidney transplant includes single or multiple kidney transplants (n = 837) and combined pancreas and kidney transplants (n = 29). ** Liver transplant includes single or multiple liver transplants (n = 459) and combined liver and kidney transplants (n = 17). *** Lung transplant includes single or multiple lung transplants (n = 298) and combined lung and kidney transplants (n = 1). **** Exact frequencies are not shown for specific transplant types due to small numbers and concern for patient confidentiality. (DOCX) [file pone.0263210.s004.docx]

| Cause of death | All transplants | Heart transplant | Kidney transplant* | Liver transplant** | Lung transplant*** |
| --- | --- | --- | --- | --- | --- |
| Cancer; N (%) | 57 (100) | <5**** | 22 (38.6) | 26 (45.6) | 6 (10.5) |
| De novo or secondary; N (%) | 37 (64.9) | <5 | 20 (35.1) | 8 (14.0) | 6 (10.5) |
| Larynx, trachea, lung, mediastinum, pleura, lip and oral cavity; N (%) | 10 (17.5) | 0 (0.0) | 6 (10.5) | <5 | 5 (8.8) |
| Esophagus, colon, liver, and intrahepatic bile duct; N (%) | 10 (17.5) | <5 | 5 (8.8) | <5 | 0 (0.0) |
| Urogenital; N (%) | 5 (8.8) | 0 (0.0) | <5 | <5 | 0 (0.0) |
| Other or unspecified type; N (%) | 7 (12.3) | <5 | 5 (8.8) | <5 | <5 |
| Relapse; N (%) | 20 (35.1) | 0 (0.0) | <5 | 18 (31.6) | 0 (0) |
| Liver and bile duct; N (%) | 16 (28.1) | 0 (0.0) | 0 (0.0) | 16 (28.1) | 0 (0.0) |
| Other type; N (%) | <5 | 0 (0.0) | <5 | <5 | 0 (0.0) |
| Infection; N (%) | 52 (100) | <5 | 21 (40.4) | 10 (19.2) | 20 (38.5) |
| Bacterial | 15 (28.9) | 0 (0.0) | 10 (19.2) | 3 (5.8) | 2 (3.9) |
| Viral | 9 (17.3) | 0 (0.0) | <5 | <5 | <5 |
| Fungal**** | 7 (13.5) | 0 (0.0) | <5 | <5 | <5 |
| Multiple, protozoal, or unspecified agents | 21 (40.4) | <5 | 8 (15.4) | <5 | 10 (19.2) |
