# Supplementary figures and images for: Trends in underlying causes of death in solid organ transplant recipients between 2010 and 2020: Using the CLASS method for determining specific causes of death
Source: PLoS One. 2022 Jul 25;17(7):e0263210. doi: 10.1371/journal.pone.0263210 (PMC9312393; doi:10.1371/journal.pone.0263210)

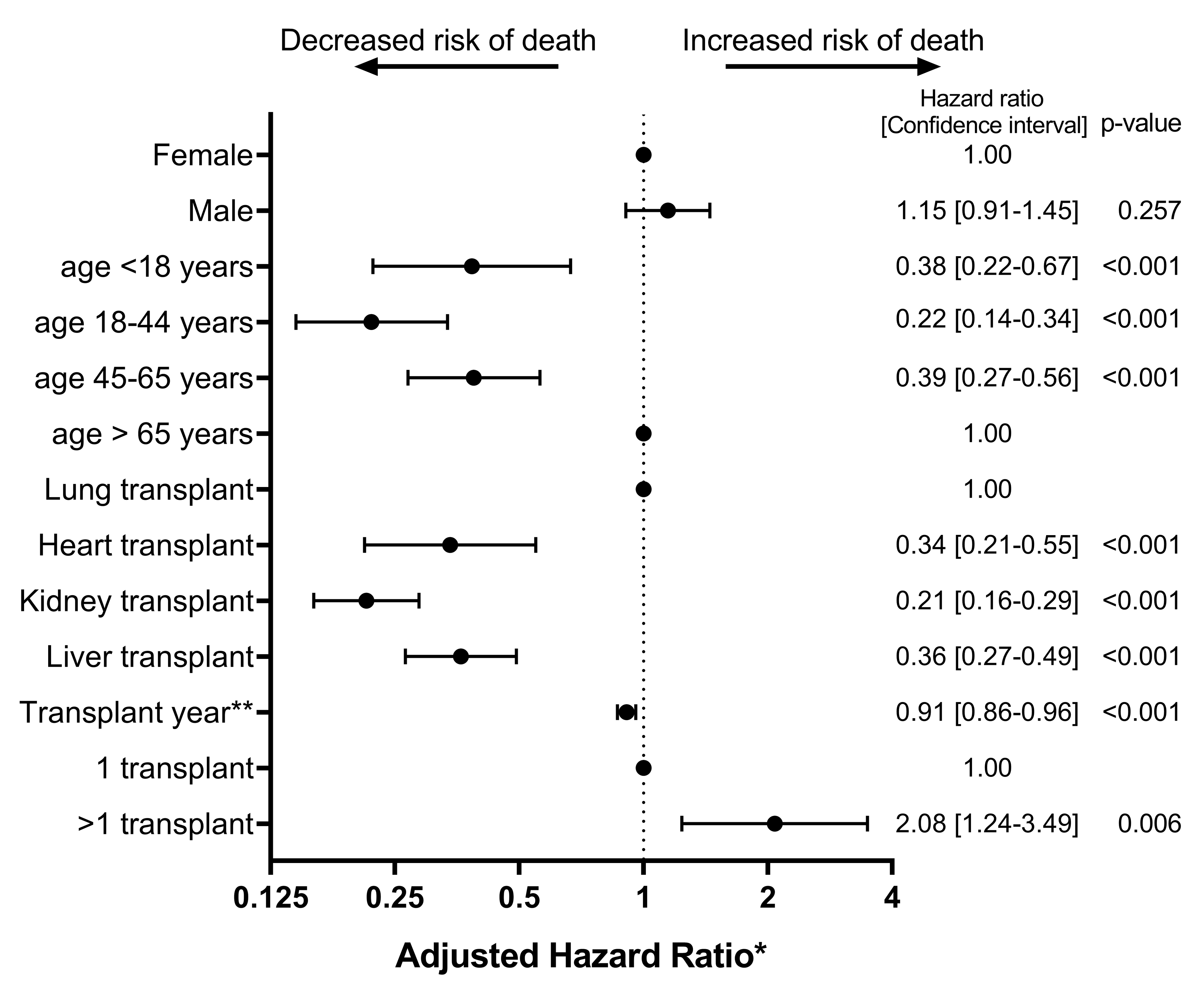

Supplement: S1 Fig — *Results are shown on a log(2)-scale. **Per one year increase in transplant calendar year. (TIF) [file pone.0263210.s005.tif]

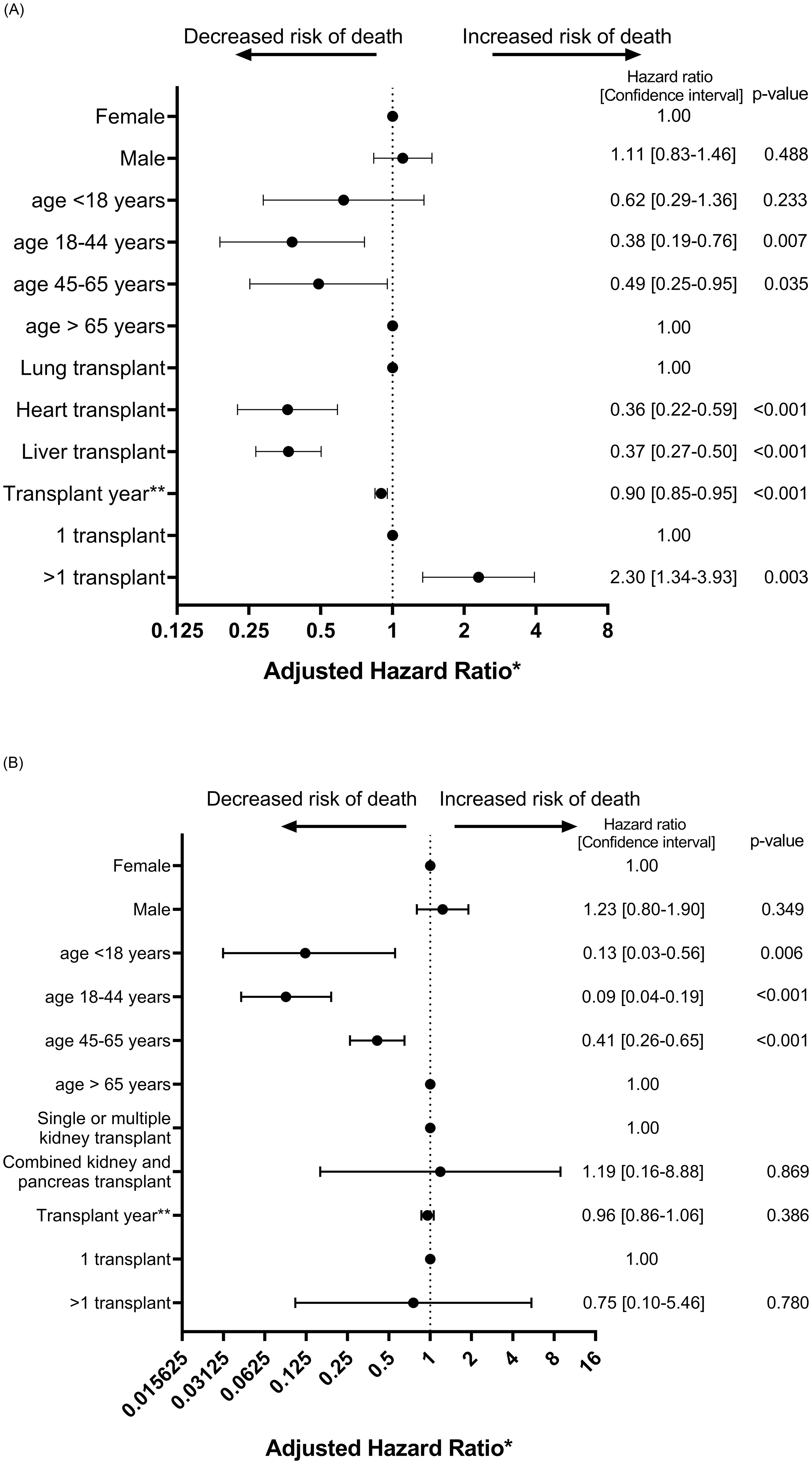

Supplement: S2 Fig — (TIF) [file pone.0263210.s006.tif]

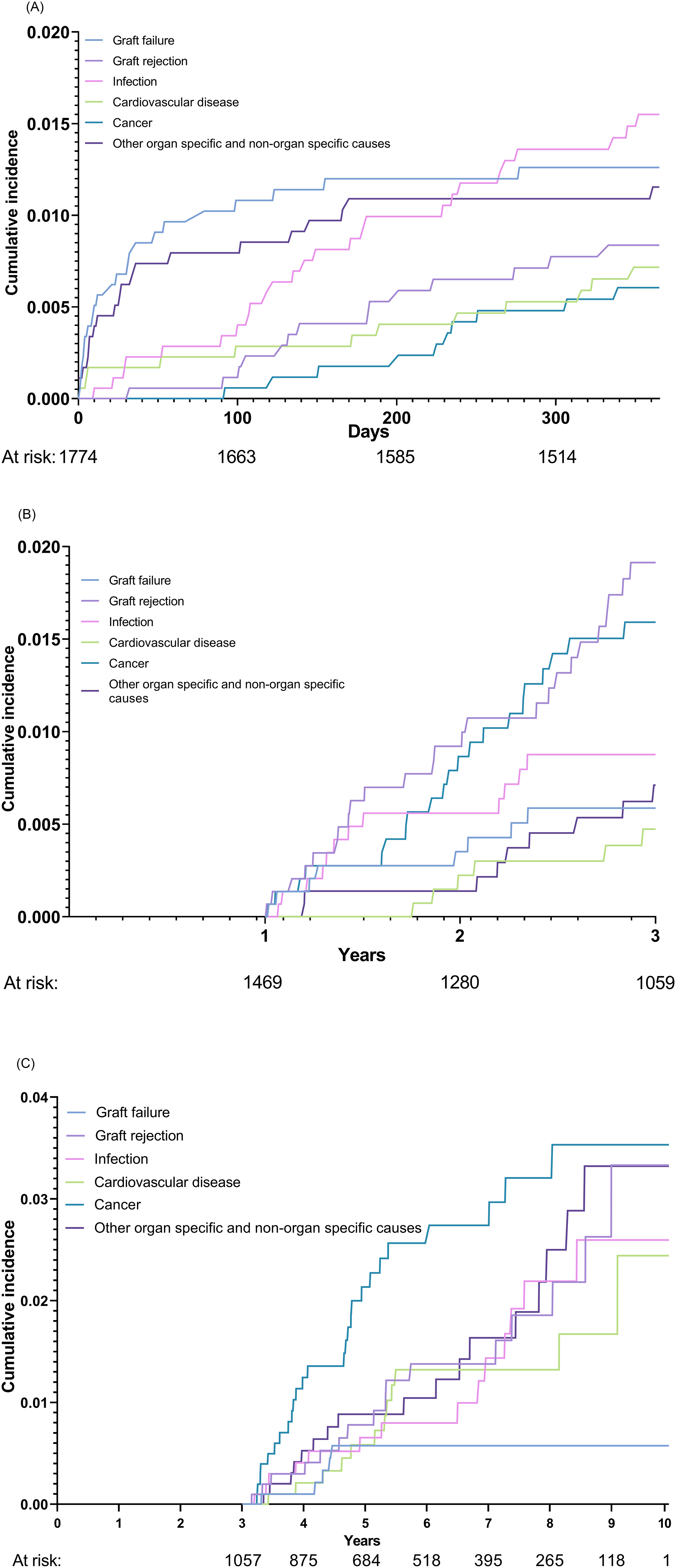

Supplement: S3 Fig — (A) ≤1 year posttransplant, (B) between >1 and 3 years posttransplant, (C) between >3 and 10 years posttransplant. *Graft rejection includes `from organ failure or dysfunction not caused by graft rejection, graft failure, cancer or infection, death from hemorrhage and death from other causes. (TIF) [file pone.0263210.s007.tif]
